# ‘Improving Health through Reducing Stress’: Parents’ Priorities in the Participatory Development of a Multilevel Family Health Programme in a Low-Income Neighbourhood in The Netherlands

**DOI:** 10.3390/ijerph18158145

**Published:** 2021-07-31

**Authors:** Gerda Wink, Gerdine Fransen, Merel Huisman, Sandra Boersma, Lieke van Disseldorp, Koos van der Velden, Annemarie Wagemakers, Maria van den Muijsenbergh

**Affiliations:** 1 Department of Primary and Community Care, Radboud University Medical Centre, 6500 HB Nijmegen, The Netherlands; Gerdine.Fransen@radboudumc.nl (G.F.); merel.huisman19@gmail.com (M.H.); Sandra.Boersma@radboudumc.nl (S.B.); liekedis@live.nl (L.v.D.); Koos.vanderVelden@radboudumc.nl (K.v.d.V.); Maria.vandenMuijsenbergh@radboudumc.nl (M.v.d.M.); 2 AMPHI Academic Collaborative Centre, 6500 HB Nijmegen, The Netherlands; 3 Wink Works for Health–Research, Projects & Training, 6708 TR Wageningen, The Netherlands; 4 Health & Society Group, Wageningen University & Research, 6700 EW Wageningen, The Netherlands; annemarie.wagemakers@wur.nl; 5 Pharos, The Dutch Centre of Expertise on Health Disparities, 3507 LH Utrecht, The Netherlands

**Keywords:** health inequities, low-income neighbourhood, perceptions of health, family health, community engagement, multilevel intervention, programme development, participatory action research, intervention logic, health promotion

## Abstract

In order to reduce health inequities, a socio-ecological approach and community engagement are needed to develop sustained interventions with a positive effect on the health of disadvantaged groups. This qualitative study was part of the development phase of a community health promotion programme. The study aimed to provide insight into the perceptions of parents in a disadvantaged neighbourhood about health, and their priorities for the community health programme. It also described the process of integrating these perceptions in the development of a multilevel plan for this programme. Participatory methods were applied to enable the engagement of all groups involved. Ten parents from a low-income neighbourhood in the Netherlands participated in five panel sessions. Parents’ priorities for improving family health were reducing chronic stress and not so much healthy eating and physical activity. They prioritised solutions to reduce their financial stress, to provide a safe place for their children to meet and play and to establish good quality communication with authorities. The programme development process resulted in objectives in which both parents and professionals were willing to invest, such as a safe playground for children. This study shows that target population engagement in health programme development is possible and valuable.

## 1. Introduction

Socio-economic health inequities persist in the Netherlands as well as throughout the world [1,2,3]. This means that, the more disadvantaged people are in terms of income and education level, the more likely they are to die sooner and to spend less of their shorter lives in good health [4,5]. In the Netherlands, the differences in life expectancy and healthy life expectancy between the highest and the lowest income and education group are seven and eighteen years, respectively [3]. Policymakers at global, national and municipal level have expressed the need for evidence to inform effective strategies to reduce health inequities [3,6,7].

Strategies that aim to reduce health inequities need to include multilevel interventions [8] and community engagement [9]. The need for multilevel interventions is underpinned by the socio-ecological perspective [8,10,11]. In this perspective, health is viewed as a function of individuals and of the environment in which these individuals live. A critical limitation of many health interventions is that they target single or individual-level factors, without simultaneously targeting other components within the individual’s environment that may influence a health concern [12,13,14]. An individual’s health is influenced by factors at intrapersonal, family, organisational, community and policy level [13,15]. Individuals’ interactions within and between these levels in their environment need to be considered to develop effective multilevel interventions [8,13].

The engagement of the community—individuals from different levels in the social system—in the development of a health intervention can contribute to understanding interactions within and between levels. Engagement encompasses a continuum of approaches ranging from information and consultation to participation and empowerment [9]. Community engagement has proved effective in improving health behaviours, health outcomes, self-efficacy and perceived social support [9]. These positive outcomes can be explained by the opportunity offered to community members to give essential input on their needs and possibilities [8,16,17]. In addition, community engagement can enhance mutual support and collective action and therefore result in an empowered community [9], which can in turn sustain the intervention [13,18].

To establish such community engagement, professionals involved (e.g., health promotion professionals) need specific skills and knowledge [8] on how to engage and partner with disadvantaged groups and organisations in a community. This is a complex task, as these groups may have different values, goals and life experiences [8] and different norms and needs than professionals in organisations [19]. These differences can result in different perceptions of health issues and possible intervention strategies [20]. Thus, when a multilevel health intervention is being developed, one of the urgent questions is how to bring different perceptions of disadvantaged groups and professionals together in a plan and how to incorporate these perceptions in the selection of objectives and activities. Disadvantaged groups’ perceptions of their individual health have been described [21,22]. However, in line with the socio-ecological perspective, individual health is also influenced by factors at family and community level [13]. What disadvantaged groups perceive as family health within their community and what they consider to be priorities for improving health are not yet known.

To facilitate the integration of disadvantaged groups’ perceptions and ideas in the development of a multilevel intervention to reduce health inequities, this study aims to provide insight into (1) a disadvantaged group’s perceptions of (a) community family health, (b) their priorities for improving health and (c) related intervention activity ideas and (2) the process of integrating these perceptions in the development of a multilevel intervention plan.

## 2. Methods

### 2.1. Study Design

This study can be characterised as community-based participatory action research (PAR), a well-proven method to facilitate community engagement [19,21,23]. In this study, PAR was combined with intervention mapping (IM) [8]. IM is grounded in community-based participatory research methods and provides a protocol for effective decision-making for intervention development [15]. IM takes the socio-ecological perspective to understand health problems and intervene at multiple levels, thereby guiding the development of multilevel interventions [15].

### 2.2. Study Context and Setting

The northern part of Wijchen was chosen as a low-income intervention neighbourhood (Box 1 describes this community’s characteristics). A family health programme ‘SameNoord Wijchen’ (‘Together Northern Wijchen’) was set up based on the need of municipal health policymakers for a policy that targeted health inequities [7]. The programme was financed by the private Dutch foundation Fonds NutsOhra (FNO) (August 2015–May 2020). Both FNO and the municipality prioritised the improvement of the self-assessed health of families disadvantaged by low income and low education, with the highest potential health gains. Families, as the prioritised target group, are an effective entry level for health interventions [13].

Box 1 **Characteristics of the northern Wijchen community, the Netherlands.**

This study took place in Wijchen, a Dutch municipality with 41,000 inhabitants. In the Netherlands, municipalities are responsible for public health services (Municipal Health Services) and for (costs of) psychosocial care and support for their vulnerable inhabitants (e.g., elderly, youth and chronically ill people). To cover the costs of medical care, all Dutch citizens have mandatory healthcare insurance and are registered with a general practitioner who acts as gatekeeper to the healthcare system. The northern part (14,935 inhabitants) of the Wijchen municipality was chosen as the intervention neighbourhood and community place in this study, as this part of Wijchen had the highest percentage of adults disadvantaged by a low income and a low education level (21%), compared to its southern (12%) and its outer (15%) part. Of the 2450 households with children in this northern neighbourhood, approximately 1300 households had a low income. In addition, 24% of the children of low-income parents received professional psychosocial support [24]. Of all inhabitants, 6.5% are of non-European migration background (https://allecijfers.nl/buurt/wijchen-noord/ accessed on 16 July 2021).

**Figure 1:**
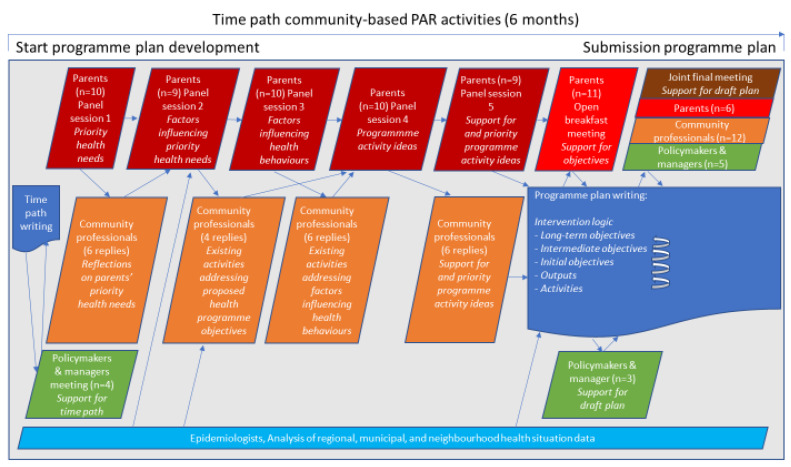
Time path of PAR activities in the programme plan development phase (August 2015–January 2016).

FNO prioritised a focus on behaviours related to smoking, alcohol consumption and overweight as important factors influencing socio-economic health inequities [25]. The Municipal Health Service, a welfare organisation, and social teams that provide care and support were involved in the programme. In this five-year programme (August 2015–November 2020), we distinguished four partly overlapping phases: development of a programme plan (6 months), development and implementation of community-based activities (42 months), programme evaluation (10 months) and writing a handbook [26] on programme development for health promotion professionals (12 months). The programme plan development phase and the subsequent activity development and implementation phase both applied a cyclical PAR process that engaged parents and professionals. This paper describes the results of the PAR process in the first programme phase (see Figure 1). The PAR-based IM process with parents and professionals resulted in an intervention logic [15]. The intervention logic is a visualisation of the hypothesised pathway by which an intervention leads to the desired programme outcomes [8,27]. In line with the IM protocol [28], the researchers explored the Dutch intervention database for possible existing evidence-based programme activities and used literature to substantiate the link between programme objectives; the outcome of this exercise informed the intervention logic.

In this PAR process, perceptions were collected and formed through interactions (the parallelograms in Figure 1) in panel sessions, meetings and e-mail contact. Most of these interactions were community engagement activities. First the parents in the low-income neighbourhood and then the community professionals were asked to express their priorities. By community professionals, we mean both professionals and volunteers attached to an organisation, who worked with families in the neighbourhood. Participating professionals included members of the social teams, social and physical neighbourhood associations, the foundation ‘Lessons in Happiness’, the ‘Healthy Wijchen’ network of health professionals and citizens, general practitioners, a youth healthcare doctor, a school director, a dietician, a community police agent and prevention advisors on addiction and on debt assistance. Policymakers, a policy advisor and managers of the programme partners formed an advisory board for the programme. In total, 46 professionals (community professionals, policymakers and managers) received e-mails with process summaries and questions, and personal conversations were held with some of these professionals.

### 2.3. Study Population

Parents living in the low-income neighbourhood were recruited for the panel by purposive sampling, through a community worker. The community worker was part of this neighbourhood’s social team. The recruiter was instructed to use the following eligibility criteria for participation: a parent living with a child in the northern part of Wijchen; no more than one participating parent per household; ability to express ideas in the Dutch language; experiencing multiple problems in the family related to finances, education, employment, health or wellbeing; or having a health disadvantage due to smoking, heavy alcohol consumption and/or being overweight. We asked the recruiter to strive for diversity in the panel participants regarding age, family composition and problems. We purposively aimed for 10–12 parents for the panel as a maximum to keep the group small enough to allow all panel participants to share their perceptions, and at the same time large enough to provide richly textured information and gain an in-depth understanding of the experiences and neighbourhood situation of disadvantaged parents. This combination of pragmatic and theoretical justifications for sample size is frequently applied in qualitative research [29]. So, we stopped recruiting after this number had been reached, which was achieved after 30 parents were approached for participation. Reasons for not participating included: not enough energy, care tasks and work. All participants were orally provided with information on the study and signed consent for audio recording of the panel sessions and anonymous reporting. They received €200 cash for their participation after the last session.

Participant characteristics were derived from oral/written questionnaires and the panel sessions.

Their educational level was described following the categories of the Municipal Health Service: low, middle and high. In line with Statistics Netherlands the low educational attainment category included: no education finished, primary education, lower or preparatory vocational education and intermediate general secondary education. (https://www.onderwijsincijfers.nl/kengetallen/onderwijs-algemeen/hoogst-behaald-onderwijsniveau accessed on 16 July 2021).

### 2.4. Data Collection by Panel Sessions with Parents

Data on parents’ perceptions and priorities for the PAR programme plan development process were collected in five panel sessions with ten parents (2015, weeks 42–49). All sessions took place in the same room in a combined primary school and community building, on the same weekday, at one- to two-week intervals and each lasted 90 min.

The topics discussed were based on IM experiences [28,30] and on the reflection log that the facilitators filled in after each session. Epidemiological data on community health and summaries of session findings were shared (orally and in writing) and discussed with the parents.

Sessions were facilitated by GW, MH and the community worker who had recruited the parents; the community worker being familiar contributed to parents’ trust and a safe atmosphere. All facilitators showed that they listened to panel members’ suggestions by repeating these verbally and writing them down. This contributed to parents’ openness. Session topics were set by the researchers and checked for agreement with parents. For instance, only after parents had confirmed their most important needs as discussed in session 1 were these further explored in session 2.

The facilitators adopted the participatory learning and action (PLA) techniques described in Table 1 to enable the democratic voicing of perceptions of those who might not readily perceive themselves as experts with valuable contributions to make [17] and to engage them in a meaningful way [31]. Furthermore, in light of the reflection log, the techniques were adapted. For instance, during the second session, the facilitators observed that plenary exchange did not meet the need for all parents to be heard, so from then on they facilitated small group dialogues [32].

The process of follow-up interaction in five panel sessions allowed for an in-depth understanding of family health needs and influencing factors, as well as for discussing everyone’s ideas for intervention activities [33]. Multiple sessions were needed to identify parents’ priority needs (session 1) as well as to dig deeper to find influencing factors (sessions 2 and 3) and to gather and prioritise programme activity ideas (sessions 4 and 5), all to feed the plan for and engagement in a programme successful in improving health.

### 2.5. Data Analysis

To acquire insight into parents’ perceptions and ideas (research aim 1), we conducted a thematic analysis in six phases [37]. All panel sessions were audio recorded (in total, 801 min), transcribed verbatim and coded. Twenty-five percent of the data were independently coded by two researchers (LD and GW) who discussed differences in coding. Next, the notes and pictures on the original flip chart papers of sessions 1 and 2 were grouped and counted to describe the family health priority themes. The described themes and sub-themes were mentioned by more than one parent on the panel, unless explicitly mentioned otherwise.

The results are presented per research aim. In the results section, quotes with quotation marks refer to written notes, by parents or the facilitator on flip chart papers and on sticky notes. In contrast, italic text with quotation marks refers to participants’ quotes from the audio recordings of the panel sessions.

The description of the process of integrating parents’ perceptions in the development of a multilevel plan (research aim 2) is based on the experience of the PAR researchers involved (GW, GF, MH) and the analysis of the baseline situation [38].

The Research Ethics Committee of the Radboud University Nijmegen Medical Centre passed a positive judgement on conducting interviews and questionnaires with (low socio-economic status [SES]) parents in this research project (File number CMO: 2017–3145).

## 3. Results

### 3.1. Panel Participants’ Characteristics

Nine of the ten panellists were female, one was male and most (*n* = 7) lived without a partner. None of the participants had a migration background. The average age was 43 (ranging from 26 to 63 years), and most of them (*n* = 7) had a low education level. All panellists lived in a household with at least one child, up to four children. Seven of the ten parents mentioned that one or more of their children suffered from psychosocial and/or physical health problems, with autism mentioned most frequently. Nine panellists confided that they experienced chronic physical and/or mental health challenges themselves. The majority (*n* = 8) depended for their income on social assistance or disability benefits, one on her partner’s salary and one person was employed. Monthly income was less than €1350 for nearly all of them (*n* = 9), and for five parents it was even €1000 or less. Some of them were dealing with debts and judicial administration.

Parents evaluated their participation in the panel sessions as ‘good, hearing many ideas, and getting information’. Parents noted it was ‘nice to think along’ and ‘good I was allowed to join, and I hope to see change in the future’.

### 3.2. Parents’ Views on What Family Health Entails in Their Neighbourhood

The panel parents mentioned several aspects of what being healthy in their neighbourhood entailed: their physical and mental health, their own and their children’s social well-being in the neighbourhood and sufficient financial means.

Physical aspects concerned being ‘free from pain’ and experiencing ‘less tiredness’. Parents mentioned healthy lifestyle behaviours such as healthy eating and physical activity, but above all they emphasised the importance of mental ‘relaxation’. No stress for parents meant ‘feeling good’, and being healthy meant ‘less stress’ and ‘avoiding stress’. They explained that their own stress affected their children and, vice versa, that their children’s stress affected them. As one parent (P4) who rarely saw her son smile said: *‘My health suffers from that too’* (P4). She wrote that, for her, health means: ‘A safe and better life for my child, without being bullied or threatened’ (P4).

Others mentioned that being healthy in the neighbourhood meant that children could play outside, but that many children in the neighbourhood sat in front of a screen and missed out on social contacts. Some parents said that they did not feel at home in their neighbourhood and missed ‘togetherness’; helping one another. They emphasised the importance of psychosocial support, which for them meant ‘being heard’, ‘valued’, ‘respected’ and ‘accepted without judgments on your size or your husband’s job’.

Sufficient financial means was another important aspect of health for parents, who agreed that not having money at all made them very unhappy. Having money enabled them to buy fresh vegetables, to eat healthily and to do sports. ‘Enjoying life’ and ‘fun’ were parts of good health, and having money enabled fun social activities to relax, like taking their children for a day out. In addition, they needed money for many forms of professional support for physical and emotional pain. Limited finances were also found to contribute to loneliness: ‘*I think there are a lot of people who are lonely. Also became lonely. Because you do not have the finances so that you can no longer go to the gym, or no longer go to the I do not know*’ (P5).

Disease was also found to cause loneliness. First, disease influenced the ability to work, and not having a job negatively affected income as well as social contacts. Besides, illness could reduce their circle of friends: ‘*You get sick, they always say then you get to know your friends. That is true. I understand that. Because if I have to go all the time to someone who constantly comes up with the same story of “I have a pain here and I have a pain there” then at a moment, you know, you will think: this time I’m going to do something fun.*’ (P2).

### 3.3. Parents’ Priorities for Improving Family Health

In line with the thoughts expressed about health, three priorities emerged from asking parents to vote for what they considered most helpful for improving the health of their family and families in the neighbourhood: (1) enough money (6 votes); (2) happy children with a safe place to meet and play (6 votes); and (3) communication with authorities (2 votes). The panel parents confirmed that these themes all related to reducing stress.

#### 3.3.1. Parents’ Priority: Enough Money

As stated, enough money enabled participants to access healthy living, physically and mentally, as well as professional support. Further exploration on what helped and what obstructed parents’ grip on their finances revealed the following factors: besides the obvious size of their income in relation to their outgoings (9 notes), parents mentioned the importance of the quality of professional guidance on managing finances (8 notes) and the negative influence of disease on finances (4 notes).

Parents indicated that more money, cheaper groceries and lower healthcare costs (health insurance premium and out-of-pocket payments) would help them to keep a grip on their finances, as healthcare costs had a large impact on the household budget: ‘*You go to the doctor and the hospital for your health, and, wham, you are paying out of your own pocket for eight months*’ (P8).

#### 3.3.2. Parents’ Priority: Happy Children with a Safe Place to Meet and Play

Most parents recognised that their children could not play safely outside in the neighbourhood because of the heavy traffic and the pond near the playground: ‘*My children have not had swimming lessons; I cannot afford it*’ (P3). Some parents added the aspect of social and mental safety, which related to not being bullied: ‘*When she went outside, she was bullied, ignored, or not allowed to join in*’ (P6).

To establish ‘a safe place to meet and play, also for children who are different’, parents suggested ‘weekly activities at the community centre’ and appreciated hearing from the facilitators that this already existed (in the panel session room for 20 years).

To improve the understanding and acceptance of children’s social disabilities by school and parents, parents suggested ‘better listening to the children in school’ as well as providing ‘more information on what it means to have a child with a disability’.

#### 3.3.3. Parents’ Priority: Good Communication with Authorities

Many parents experienced a limited ability of authorities to help them, which parents related to (1) professionals’ limited ability to listen; (2) limited communication among different authorities; and (3) limited clarity of communication by authorities. They felt that instead of listening, authorities ‘draw their own plan’ or ‘think easy, as they themselves have enough income’. Parents perceived the communication with authorities as a struggle, adding to the stress that they experienced in daily life. A parent with a chronic disease explained that she moved to another municipality and that she had to struggle for 12 years to obtain the disability benefit that she used to receive. She suggested that authorities should enquire about a person’s situation at, for example, the previous municipality or health insurance, ‘*Because that also saves an enormous amount of stress*’ (P6).

Another parent, whose household income has decreased a lot as a result of a new national policy measure, would have liked the municipality ‘to stand up’ for her situation at national level.

The communications by tax authorities and the municipality were seen as obstructing the fulfilment of the parents’ need for enough money, because of the feeling of being sent ‘from pillar to post’ and difficult procedures, ‘*They also need to know that people are not all scholars*’ (P6).

In parents’ view, ‘easier formulation of forms’ and ‘competent professionals’ that really listen and ‘take people seriously’ would help to improve communication with authorities.

### 3.4. Parents’ Ideas for Programme Activities

As parents had indicated that their priorities for improving the health of families in the neighbourhood all related to reducing stress, they mentioned many helpful actions to cope with stress, such as talking about it with someone (including professional help), ending non-supportive relationships, not caring about what others think of you, laughing with friends, creating something with your hands, physical activity and changing focus and changing your mindset (for example, looking at what is possible at low cost like making pancakes with your children).

Besides these suggestions for coping with stress at an individual level, parents gave suggestions to reduce the stress of families in their neighbourhood. A group of parents was very enthusiastic about their ideas for a safe playground, as this would tackle multiple sources of stress. If there was no entrance fee, the playground would also be accessible for families with little money. Visible behaviour rules, safeguarded by volunteers, would allow for the social safety of all children, so children with a disability would also feel welcome. A fence around the playground would keep children safe from running off towards water or traffic, and benches would allow parents to get in contact with one another. Both the fence and the benches would enable parents to relax. A safe playground could also be a place to organise activities for children like a hunt for treasure hidden in the sand, which could be publicised by a flyer. Additional ideas for programme activities included:

- Information on, and easy referral to, organisations that could help to reduce financial stress, for instance by paying for swimming lessons for children. Parents indicated that many people did not make use of existing possibilities as people did not know of their existence, or did not know where or whom to ask about them, or the people that they asked did not know. Parents suggested that the authorities or the general practitioner should refer people to specific information on websites or inform them on who does what.
- Adjustment of forms from authorities to make them easier to understand, to reduce stress related to communication. Parents suggested that the municipality should accompany verbal information with written information that they could read at home at leisure. They suggested that professionals working at authorities should empathise with their situation, think along with their ideas and keep their word.
- Regular low-cost group activities for children to meet friends, differentiated by children’s age and disability, for instance, ‘playing games, playing soccer, craft making’.
- Low cost, social activities for parents to increase togetherness, for example, a healthy-cooking workshop or creative activities. However, many parents expected the participation threshold to be (too) high; for example, people would be afraid to participate alone, fearing that people going there already would all be friends. To overcome this threshold, they suggested an accompanying buddy or a high financial reward, for example, a bingo night with high value prizes for all, though this was not considered realistic.

### 3.5. Integrating Parents’ Perceptions in Programme Plan Development

**Figure 2:**
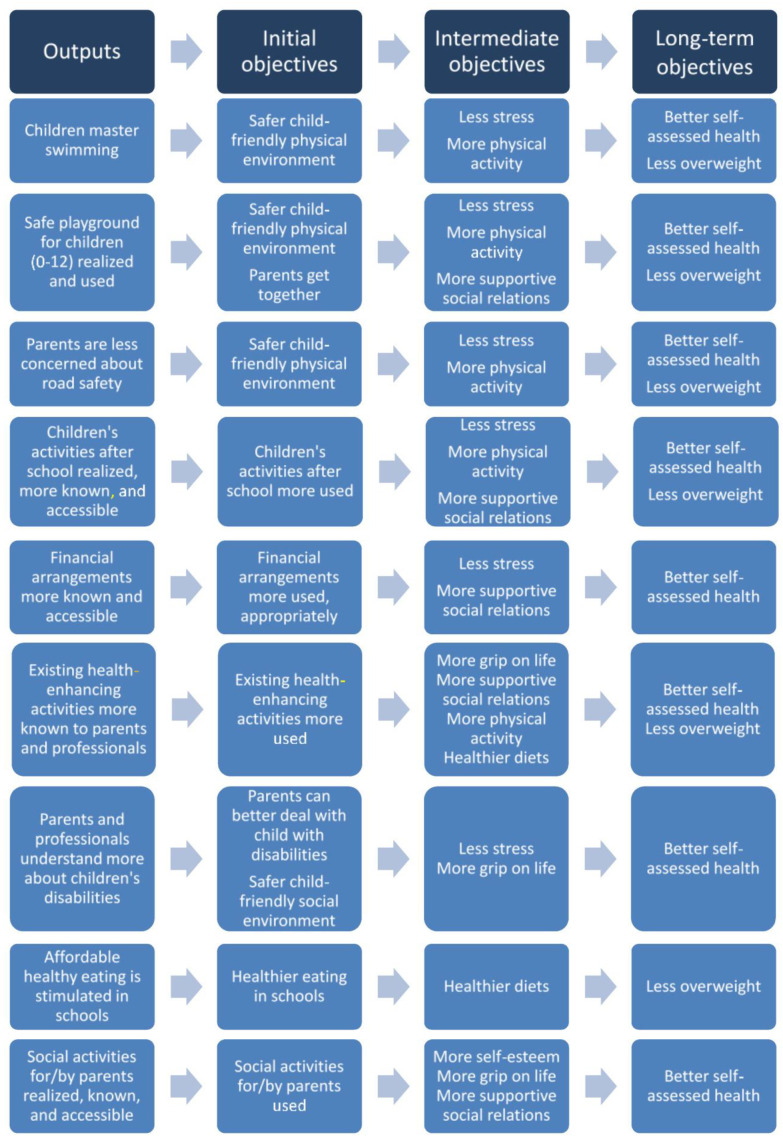
Intervention logic adapted from programme plan.

In the programme plan, parents’ experiential knowledge was integrated with practical knowledge from professionals working with families, policy knowledge, epidemiological knowledge about the community’s health characteristics (see also Figure 1) and scientific knowledge on health promotion in disadvantaged communities. This resulted in a multilevel programme plan describing the intervention logic of outputs and objectives (see Figure 2), serving as a visualisation of the hypothesised pathway of reaching programme outcomes.

Regional health data showed a high prevalence of psychosocial problems in children and of loneliness, depression (related to stress and self-assessed health), overweight and insufficient physical activity in adults (related to overweight). Consistent with this regional health data, parents considered healthy eating and physical activity more important to address than tobacco and alcohol use.

The outputs and objectives in the intervention logic reflected both parents’ priorities (social, financial and child-safety needs) and health policy priorities (healthy eating and physical activity, grip on life). To formulate the outputs and the initial objectives, the input of both parents and community professionals was essential: parents indicated which ideas, from the assembled list of programme activity ideas, they considered most relevant and to which they wanted to contribute. Community professionals replied to these same questions. Then, activities to which both parents and professionals were willing to contribute were included in the programme. For example, the first desired output (Figure 2), ‘children master swimming’, met parents’ need for safety as well as the concerns of the school director and a healthy school policy advisor. This need for swimming skills was not obvious from regional health data but was made apparent through the contribution of parents and professionals who knew the community. Support for the outputs and the objectives in the intervention logic was confirmed through checks with parents and professionals (Figure 1). After this check, the intervention logic was adapted based on the results of the PAR as described in this article, with the following additions: the objectives ‘less stress’, ‘parents get together’, ‘more grip on life’ and the outputs ‘parents and professionals understand more about children’s disabilities’ and ‘more accessible’. Moreover, outputs and initial objectives were split, to be logical.

Besides the intervention logic, the programme plan described the input and the organisation of the subsequent programme phase: parents and community professionals assigned to take part in facilitated workgroups to develop and implement activities to realise the desired outputs and objectives. Parents indicated that they needed support from the facilitators to think along in the development and in organizing activities. Parents’ suggestions for activities meeting their needs and possibilities informed the programme plan as well as programme activity development.

## 4. Discussion

### 4.1. Our Main Results

**Figure 3:**
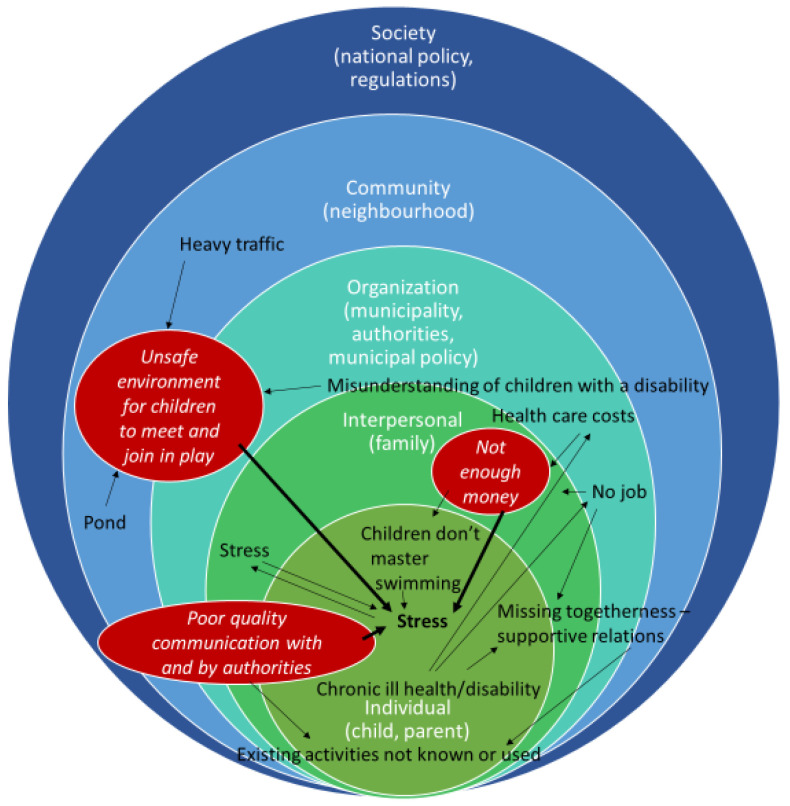
Parents’ perceptions of factors causing stress and their relatedness, positioned in the corresponding level of influence on health (Figure from [15]). Parents’ priorities for improvements are written in italics in the red ovals.

For parents in a low-income neighbourhood, family health meant above all less stress and more social well-being and safety for themselves and their children, as well as sufficient financial means. Their priorities for improving family health all related to reducing stress: they needed enough money, a safe place for their children (including those with a disability) to meet and play and good quality communication with authorities. These needs are interrelated, as they mentioned themselves (see Figure 3), resulting in their most concrete suggestion to build a safe playground, as this would tackle multiple sources of stress.

Parents’ ideas on intervention activities implied that activities needed to be financially supportive or affordable, that they should enable the building of social supportive relationships, that activities should include warm, personal guidance to enable participation, and understandable written communication to be accessible.

Integrating their priorities and ideas led to the intervention logic in Figure 2, which was supported by all those engaged.

### 4.2. Comparisons with Existing Literature

This study showed that the three major lifestyle themes in governmental public health policies (overweight, tobacco and problematic alcohol use) [39] were not among the health priorities of disadvantaged parents, but that reducing stress was. Chronic stress, for instance due to poverty, may lead to depression, increased susceptibility to infection, diabetes, high blood pressure and increased risk of heart attack and stroke [40,41,42]—diseases that are more prevalent in lower SES strata in industrialised countries [42]. Furthermore, chronic stress negatively affects the executive brain functions, leading to more short-sightedness and risk-averse decision-making (‘not willing to do something if it is possible that something bad could happen as a result’ [43]), favouring habitual behaviours at the expense of goal-directed ones [44]. All of this impedes healthy lifestyle behaviours. To improve the health of disadvantaged groups, addressing sources of stress, such as poverty, merits attention in public health policies.

Similar to the intervention activities that our study parents suggested, Ball [4] and Nagelhout et al. [33] found that intervention activities for disadvantaged households need to be free/cheap, to include social interaction and to reduce stress. Personal guidance towards socially or financially supportive activities might enable the bridging of what Ten Kate et al. [45] described as ‘feelings of cultural entitlement’, for example, not being able to apply for financial support as a consequence of the complicated language that more highly educated people use in application forms.

### 4.3. Methodological Reflections

The PAR programme development process combined with an IM protocol was effective in engaging parents in the neighbourhood to contribute to positive change for community family health. The desired outputs, initial objectives and the intermediate objective ‘less stress’ were not obvious from regional health data but apparent only from interactions with parents who knew the neighbourhood. Distinguishing outputs and objectives in the intervention logic (IM) provided structure for programme implementation and evaluation. Unlike other intervention logics (e.g., [46]) that are based primarily on theoretical models, we chose to stay close to the language of the engaged parents and professionals, as it is known from the literature that a theoretical model does not add to the effectiveness of interventions for socio-economically disadvantaged groups [47,48].

The process of developing a programme plan as described in this study can guide other PAR researchers and programme planners who want to develop a multilevel intervention with a comparable group [49]. As many interventions aiming to reduce health inequities still target single or individual-level factors [12,13,14], our experiences could feed into the much-needed development of refined guidelines for systematically designing multilevel interventions [14]. The multilevel and interrelated factors influencing the health of disadvantaged families (Figure 3) confirm the need for a systems approach.

The process of engaging disadvantaged parents in programme plan development was time-intensive; it required small steps (two to three questions per session at max) and multiple sessions. The six-month process as described in this study turned out to be too short for collaboration on SMART change objectives. Stevens et al. [49] confirmed that adequate time and resources are essential for developing multilevel interventions targeting disadvantaged populations. In practice, with limited budgets for process guidance, the required time and resources, might be hard to negotiate [28,34]. However, the chosen approach proved to be worthwhile as it enabled the parents involved to be meaningfully engaged in the development of the multilevel health improvement intervention, a process that they valued, empowered them with new ideas and has the potential to empower them for future contributions [35].

However, a potentially effective means for further engaging the parents could not be used. We aimed also to engage parents who were not able to attend the panel sessions, because of, for example, work hours or caring tasks for family members, by asking them questions through WhatsApp. Seven parents were willing to participate through WhatsApp, but this was not approved by the Medical Ethical Committee because of privacy legislation. In line with Den Broeder’s [50] recommendation to explore the wider engagement of disadvantaged groups through making use of mobile applications in data collection, we suggest that researchers explore judicially acceptable ways to use WhatsApp or another messenger app for data collection.

### 4.4. Strengths and Limitations

The perceptions found are specific to the participating parents in northern Wijchen, who are disadvantaged by living in a low-income neighbourhood, with a low household income, nearly all with a (chronic) health condition and most with a low education. Parents with middle and higher income and education levels (approximately 1150 of the 2450 households living with children in this neighbourhood [24]) might have different perceptions, priorities and ideas.

Ten parents is a small number. Therefore, the programme plan was also based on neighbourhood health monitor data from more parents and children, as well as on the insights of professionals working with multiple families. Programme follow-up research involved 124 neighbourhood parents through oral/written questionnaires.

Parent panel recruitment yielded only one male participant; two other male parents were not able to participate because of work hours. Recruitment was also not inclusive of parents who did not speak the Dutch language or did not have enough energy to participate. Noticeably, many parents mentioned having a child with autism. This can be explained by the recruitment strategy of approaching parents who had sought assistance from the social team, which also provided psychosocial support for children. Trentacosta et al. [51] explained that economic stressors can affect parenting strategies adversely, contributing to behavioural difficulties in children with autism [52]. Moreover, caring for a child with autism can affect the caregiver’s earnings and employment status. These effects are often especially profound for mothers of children with autism [53], who, on average, earn 56% less than mothers of typically developing children [54].

### 4.5. Recommendations

#### 4.5.1. For Health Policymakers

We suggest that municipal health policymakers engage with disadvantaged inhabitants to understand their experiences and priorities. Furthermore, we would advise that they align with policymakers in the socio-economic field, such as debt assistance and poverty alleviation, given the interrelatedness of health and social determinants (Figure 3).

#### 4.5.2. For Further Research and Research Funding

Additional research needs to investigate further programme outcomes over the entire programme period, as well as the related mechanisms and influencing contextual factors. Vaandrager et al. [55] evaluated 46 programmes and found that the 19 programmes (out of 46, including the programme in this study) that got six months funding to conduct a situation analysis and develop the programme with disadvantaged families were more likely to find positive changes in overweight and self-assessed health. Most programmes needed at least a year for programme development with families and finding an appropriate evaluation approach. Therefore, with Vaandrager et al. [55], we suggest investing a lot of time in programme development and financing it.

## 5. Conclusions

Disadvantaged parents’ priorities for improving family health in the neighbourhood are all focused on reducing chronic stress and not so much on healthy eating and physical activity. This outcome underpins the need for the involvement of the target population in the development of health promotion interventions—an involvement that has been shown in this study to be possible and valuable. Therefore, municipal health policymakers should engage with disadvantaged citizens to understand the interrelatedness of factors influencing their health and their priorities to make positive changes. These include understandable and accessible information as well as affordable interventions aimed at increasing child safety and social contacts.

## Figures and Tables

**Table 1:** Panel session topics, questions and techniques.

Panel Session	Topic	Questions to Parents	Techniques
1	Family health needs that parents prioritised to be addressed in the programme	1. What does being healthy in your neighbourhood mean for you and your family? 2a. What aspect of your own or your family’s health would you like to see improved? 2b. What is the most relevant need for (i) your own family and (ii) families in the neighbourhood?	Co-generated ground rules (PLA [34]) Flexible brainstorming (PLA [35]) Commentary chart (PLA, [34]); parents explained pictures and ideas on sticky notes and the facilitator assigned these to a relevant dimension of the positive health concept [36] on this chart Direct ranking (PLA [35]) of family health needs, using stickers to vote
2	Factors influencing three priority health needs emerging in session 1 Lifestyle themes prioritised by parents	1. What are the factors that facilitate or obstruct the improvement of family health for priority needs? 2. Which of the healthy lifestyle themes that the health organisations think are important (smoking, alcohol use, or healthy eating and physical activity) do you consider most relevant for families in the neighbourhood to address?	Flexible brainstorming (PLA [35]) on facilitating and obstructing factors for three priority health needs Cap exercise to check synthesis of first session and to prioritise lifestyle theme: quit smoking (put your cap in the air), healthful eating and physical activity (cap on head) or reduce alcohol use (cap on table)
3	Factors influencing health behaviours	1. What does healthy eating mean for you, and what do you need to eat more healthily, a. for you as parent? b. for your child(ren)? 2. What do you and your children need to be more physically active? 3. What helps you to relax in stressful periods?	Carousel [30] of small group dialogues in three rounds. The three facilitators switched subgroups after 15 min, taking the flip chart paper with notes to the next subgroup. The subgroup facilitators presented a summary of findings to all parents.
4	Ideas for programme activities for health behaviours	1. What are your ideas for solutions/programme activities for a. healthy eating, b. physical activity, c. relaxation?	Carousel [30] of small group dialogues in three rounds. In this session, the parent subgroups changed tables after rounds 1 and 2.
5	Programme activity ideas prioritised by parents	1. What activity will contribute most to family health in your neighbourhood? 2. In what activity do you see your family participating? 3. To which activities would you contribute?	Voting with stickers for priority programme activities Two small group dialogues on questions Plenary brainstorming session on implementation of activity ideas Speed evaluation (PLA [35]) on experiences in the panel meetings

PLA: Participatory learning and action.

## Data Availability

Please contact the corresponding author in case of a research related request.
